# Bioactive Molecules from Tropical American Plants: Potential Anti-Inflammatory Agents for Cytokine Storm Management

**DOI:** 10.3390/molecules30071486

**Published:** 2025-03-27

**Authors:** Erika Plazas, Lucellys Sierra-Marquez, Jesus Olivero-Verbel

**Affiliations:** Environmental and Computational Chemistry Group, School of Pharmaceutical Sciences, University of Cartagena, Cartagena 130014, Colombia; eaplazasg@unal.edu.co (E.P.); lsierram@unicartagena.edu.co (L.S.-M.)

**Keywords:** cytokine storm, hyperinflammation, natural products, polyphenols, alkaloids, terpenoids

## Abstract

The cytokine storm, a hyperinflammatory response characterized by the excessive release of pro-inflammatory mediators such as TNFα, INFγ, IL-1β, IL-6, and GM-CSF, has been identified as a critical factor in the progression and severity of acute inflammatory conditions. Regulating these pathways is essential for mitigating systemic damage and improving outcomes. Natural products from tropical American plants have shown significant potential in modulating these hyperinflammatory responses. Key polyphenols, like quercetin and luteolin, found in plants such as *Achyrocline satureioides* and *Mangifera indica* demonstrate the downregulation of NF-κB and inhibition of pro-inflammatory cytokines. Alkaloids, such as berberine and mitraphylline, isolated from *Berberis* species and *Uncaria tomentosa*, respectively, have shown potent effects in suppressing nitric oxide production and regulating inflammasomes. Terpenoids, including parthenolide from *Tanacetum parthenium* and curcumol from *Curcuma longa*, exhibit multitarget activity, reducing cytokine levels and inhibiting key inflammatory enzymes like COX-2 and iNOS. These findings highlight the immense potential of bioactive compounds from tropical American plants as modulators of immune–inflammatory pathways, providing a foundation for developing effective therapeutic agents to counteract the severe effects of cytokine storms.

## 1. Introduction

Infectious agents constitute one of the most challenging and high-risk global health problems. Even though, the healthcare system and medical research have evolved in recent decades, emerging and re-emerging infectious agents still threaten human health [1]. Accordingly, since 2015, the World Health Organization (WHO) has listed priority infectious diseases that have potential risks to generate a public health emergency [2]. For example, in the 2018 WHO’s Blueprint, different diseases caused by viruses such as Ebola, Zika, Middle East respiratory syndrome (MERS), and severe acute respiratory syndrome (SARS) were cataloged as some with the highest probability of epidemics due to their elevated transmission rate, insufficient prevention and diagnosis, and lack of treatments [3]. However, despite the WHO’s earlier warnings, in 2019, a new strain of human coronavirus (SARS-CoV-2) linked to severe respiratory syndrome cases in China was reported; this disease was named COVID-19 in February 2020 [4]. Different studies suggest that SARS-CoV-2 began in animals, probably bats, and then was transmitted to humans, becoming the seventh human coronavirus. Later, the pathogen could have mutated and adapted to acquire human-to-human transmission, enabling its fast expansion in China [5]. In addition, SARS-CoV-2 is highly contagious compared to other SARS-CoV, which facilitated its quick spread to other countries worldwide; thus, on 11 March 2020, COVID-19 was declared a pandemic by the WHO, the second pandemic of the 21st century after swine flu in 2009 [6]. According to the WHO COVID-19 situation report, by December 2024, more than 121 million confirmed cases and near to 2.7 million deaths were reported [7]. Undoubtedly, in the last year, the world has faced an exceptional challenge due to the devastating consequences at the social and economic levels. The COVID-19 outbreak challenged not only the health system and worldwide economy but also the pharmaceutical industry and research groups around the world, who provided a number of research and development (R&D) studies and reported candidates from clinical trials. For instance, the pipeline of clinical trials related to COVID-19 increased from approximately 500 trials in April to more than 4000 in September compared to the previous year, with small molecule and biological (Bios) trials composing nearly 50% of the total [8,9]. As a result of these remarkable fast-tracked research programs, in December 2020, the Pfizer–BioNTech vaccine was approved in the United Kingdom, and just over one week later, it was authorized for emergency use by the United States Food and Drug Administration (FDA) [10,11]. Notwithstanding all the important advances achieved after the pandemic was declared, it is still necessary to keep searching for therapeutic options, in particular, to treat its severe pathologic events [12,13,14,15,16,17].

The exact mechanisms involved in the pathophysiology of COVID-19 have not been fully elucidated. Nonetheless, at the clinical level, a wide spectrum of symptoms ranging from mild respiratory illness and pneumonia to severe multiorgan failure have been recognized [18,19]. Furthermore, it is known that the inflammation pathway linked to the immune response plays a crucial role in the progress and severity of the infection. Different scientific evidence has pointed out that exacerbating pro-inflammatory cytokines levels trigger an imbalance between protective and altered immune response, contributing to disease severity and multiorgan damage [20,21]. Hence, targeting the hyperinflammatory pathway and cytokine storm constitutes a promissory strategy to control infectious diseases related to cytokine storms.

Historically, humans have used nature as a source of medicinal preparations for the treatment of multiple illnesses. This has not been in vain as plants are the basis of traditional medicine from different cultures around the world and are still providing bioactive compounds for drug discovery [22,23]. Plant-derived compounds constitute a promising source of anti-inflammatory agents with a high scaffold diversity and a large number of mechanisms of action, including the modulation of pro-inflammatory cytokines, inhibition of key enzymes involved in inflammatory cascades, and regulation of prooxidative species [24,25]. Therefore, this review summarizes studies on peer-reviewed articles available in Google Scholar (https://scholar.google.com), PubMed (https://www.ncbi.nlm.nih.gov/pubmed), Scopus (https://www.scopus.com), and ScienceDirect (https://www.sciencedirect.com). The search strategy consisted of using the keywords “natural products”, “inflammation”, and “tropical plants”, combined with words associated with different aspects of *in vitro* or *in vivo* activity, molecular targets, and structural motifs, all of them starting points in the search for new sources of therapeutic agents for the treatment cytokine storm-related diseases.

### 1.1. Hyperinflammatory Response and Cytokine Storm

Clinical and scientific evidence have shown that patients with acute COVID-19 present high levels of pro-inflammatory cytokines as a result of an abnormal immune response [26]. The systemic inflammation response, also denominated “cytokine storm”, leads to the over-activation of inflammatory cytokine production and high levels of immune cells, which have been suggested to contribute to disease severity and a high risk of mortality [20]. Postmortem studies of lung tissues revealed high amounts of T cells, such as Th-17 and CD8^+^, supporting the fact that the overactivation of an innate and adaptive immune response can trigger specific cascades and promote an inflammatory response [21]. In addition, it has been found that levels of CD8, CD4, and NK cells are reduced in the peripheral blood of COVID-19 patients. This affection has also been associated with the progression and severity of the infection [27]. Therefore, understanding the immune–inflammatory response generated by SARS-CoV-2 infection is a keystone to the search for effective therapeutic options for the cytokine storm.

The progression of the infection in COVID-19 occurred in different stages, including the viral entry in lung cells, viral propagation causing lung injury, and hyperinflammatory cascade with a cytokine storm [28]. Once the virus has infiltrated respiratory cells, it can replicate and infect more cells, leading to damage-associated molecular pattern (DAMP) release; subsequently, DAMPs are identified by recognition receptors, which, in turn, activate the production of pro-inflammatory cytokines [29]. Indeed, in asymptomatic cases, after DAMP recognition, type I and III interferons (IFNs) are secreted, and virus replication is inhibited. Nevertheless, in patients whose IFN production is reduced, either because of existing comorbid conditions or dysfunctional immune response, there is a subsequent arrest of inflammatory cells, predominantly macrophages and monocytes [21]. Those cells have been demonstrated in SARS-CoV-2 infection and present an exacerbated production of cytokines, primarily IL-6, IL-1β, MCP-1, IFN-γ, and TNF-α, among others [30]. Other evidence suggests that TNF-α plays a key role as an inflammation mediator in COVID-19, deregulating signaling cascades, such as the activation of the transcription factor nuclear factor-κB (NF-κB), and favoring the overproduction of other pro-inflammatory cytokines, such as IL-1 and IL-6 [31]. Moreover, the abnormal activation of NF-κB has been demonstrated to be associated with pro-oxidative pathways due to an excess of reactive oxygen species (ROS) leading to apoptosis in different tissues [32].

Considering the aforementioned evidence, all the pathways that might allow regulation of the hyperinflammatory response and cytokine storm can be considered potential druggable targets to avoid the pathological events observed in severe cases of SARS-CoV-2 and reduce mortality associated with the disease. Therefore, natural products that have been shown anti-inflammatory activity by the downregulation of pro-inflammatory cytokine production, inhibition of NO and/or ROS production, and regulation of transcription factors, among others, would be promising in the search for effective and more accessible therapeutic options for acute cases of COVID-19.

### 1.2. Plant-Derived Natural Products as a Source of Anti-Inflammatory Agents

Plant-derived natural products (NPs) have played a critical role in drug discovery as a source of small molecules with unique advantages. For instance, according to the US Food and Drug Administration (FDA), about 34% of the total small chemical entities approved between 1981 and 2010 were derived or related to them [33,34]. Despite the emergence of cutting-edge technologies and the omics applied to drug development, natural product-based drug discovery still presents different advantages, and it is worth highlighting NPs’ structural diversity and metabolite-likeness characteristics [33]. In fact, due to the evolutionary role of NPs, these compounds are provided with unique structural diversity, covering a relatively large portion of the chemical space, with a great variety of scaffolds and pharmacophores [35]. Furthermore, since NPs are biosynthesized in pathways catalyzed by enzymes, they possess the minimal structural features to be recognized and transported by bio-macromolecules; thus, NPs are not only potentially bioactive but also can be recognized by transporters to cross membranes and reach the site of action [33,36].

In addition to the above-mentioned advantages of NPs, there is vast evidence of the ancestral use of botanical preparations to ameliorate inflammatory processes linked to different diseases, such as pain, rheumatism, arthritis, and fever, among others [37,38,39]. Turmeric (*Curcuma longa*) is one such example. Its preparations have been used for the treatment of chronic inflammatory diseases in the folk medicine of different cultures. Although its traditional medicinal uses date back nearly 4000 years in Ayurvedic medicine mostly for digestive disorders, as cultivations of this species expanded throughout the world, new uses emerged, including the prevention and treatment of inflammatory conditions [40]. In addition, *in vitro* studies have supported the traditional anti-inflammatory use of turmeric. For example, in 2005, it was reported that its organic (DCM–methanol) extract presents the inhibition of TNF-α and prostaglandin E2 (PGE2) production in HL-60 cells after exposition to 1 mg/mL lipopolysaccharide (LPS), with IC_50_ values in the micromolar range [41].

The American tropics is the region with the greatest diversity of vascular plants worldwide, with almost a third of total species and more than twice as many plants reported for Southeast Asia and the Afrotropical region [42]. Therefore, the enormous biodiversity of the Latin American tropics constitutes an invaluable source of bioactive natural products that can be the basis for the development of new therapeutic agents. As well as in other regions worldwide, the folk medicine of different communities in Latin America uses plants for the treatment of inflammatory pathologies, such as rheumatism, arthritis, chronic pain, and cardiovascular diseases, among others [38,43,44]. In fact, there have been some successful cases in the approval and commercialization of phytotherapeutics based on ethnopharmacology. In Brazil, a product known as *Acheflan* was patented and launched by the pharmaceutical company Aché^®^ (Guarulhos, Brazil) for the treatment of chronic muscle pain. This phytomedicine mainly contains the essential oil of the medicinal plant *Cordia verbenacea* DC (Boraginaceae), commonly known as “maria milagrosa” and traditionally used for the treatment of inflammatory conditions in Brazil [43]. Consequently, based on ancestral knowledge of the tropical American plants, *in vitro* and *in vivo* studies have been carried out to determine their inflammatory potential. Some selected tropical plants that stand out due to their promissory anti-inflammatory or immunomodulatory activity and that might be useful in the search for therapeutic options against a hyperinflammatory response to COVID-19 are summarized in Table 1.

Different Asteraceae species endemic to the tropical regions of South America have shown immunomodulatory and anti-inflammatory properties. For example, *Achyrocline satureioides* (Lam.), also popularly known as “Marcela”, has been used by different Indigenous tribes for the treatment of respiratory and viral infections and inflammatory diseases. *In vitro* and *in vivo* studies have supported folk medicine uses [50]. Indeed, aqueous infusions of the aerial parts have been demonstrated to regulate the production of the pro-inflammatory cytokines IL-4 and IFN-γ, as well as the inhibition of ROS production in human peripheral blood mononuclear cells (PBMCs) and polymorphonuclear leukocytes (PMNs) [46]. Also, *Baccharis* species widely distributed in the Andean region of South America are characterized by their anti-inflammatory uses. This popular knowledge has been substantiated by *in vitro* studies that have shown the modulation of signaling pathways (NO), enzyme inhibition (COX-1, 5-LOX), and regulation of transcription factors (TNF-α) involved in inflammatory processes [51].

Fabaceae (Leguminosae) is the plant family with the highest number of species in the American tropics, mostly distributed in tropical rain and dry forests, which include a high diversity of species at morphological, ethnobotanical, and chemical levels. About 500 species are recognized for their therapeutic properties and have been included in traditional medicine systems around the world [52]. In the Amazon region, exudates obtained from the *Copaifera* species, also known as *Copaiba* oils, are used by the Indigenous peoples of the region as topic anti-inflammatories [53]. The medicinal properties of copaiba oleoresin have become popular in recent years, and different scientific evidence has reinforced its anti-inflammatory and anti-cancer effects. In fact, oleoresin from *Copaifera multijuga* has shown potent anti-inflammatory effects in both *in vitro* and *in vivo* models, reducing NO and pro-inflammatory cytokine production [53] (Table 1).

In a recent study, different medicinal species from the Colombian Caribbean region used in folk medicine for the treatment of inflammatory and respiratory conditions were screened *in vitro* to determine their anti-inflammatory potential [49]. In the preliminary analysis with RAW 264.7 macrophages stimulated with LPS, the ethanolic extracts of *Croton malambo* (Euphorbiaceae) and *Physalis angulata* (Solanaceae) exhibited inhibition of nitric oxide (NO) production (Table 1). Further studies with *P. angulata* revealed that active metabolites are mainly present in the dichloromethane fraction by inhibition of the production of key pro-inflammatory mediators, such as interleukin (IL-1β), prostaglandin E_2_ (PGE_2_), and tumor necrosis factor (TNF-α), which supports the anti-inflammatory potential and ethnopharmacology use of *P. angulate* [49].

On the other hand, some species of tropical fruits, such as *Mangifera indica* (mango), *Persea americana* Mill (avocado), *Anacardium occidentale* L. (cashew), and *Tamarindus indica* (tamarind), have also been studied in the inhibition of pro-inflammatory mediator production, finding positive anti-inflammatory effects with several parts of the plant (leaves, bark, or fruits) (Table 1). Such findings suggest that it would also be plausible to take sustainable advantage of cultivated edible species using different parts for medicinal purposes, which would contribute to the preservation of biodiversity.

As shown in Table 1, previous anti-inflammatory studies of tropical American plants have revealed their potential in the regulation of key signaling pathways involved in the immune–inflammatory response. Indeed, it is possible to outline that several plant extracts can downregulate the NF-κB signaling cascade that is recognized to induce the gene expression of inflammatory cytokines and accelerate the production of ROS in SARS-CoV-2 infection [32]. Therefore, the examples selected in this review point out that ancestral knowledge and traditional uses of plants constitute a strong pillar in natural product-based drug discovery, and this should not be the exception in the current pandemic. Therefore, it is consistent to draw on either ethnobotanical knowledge or previous anti-inflammatory activity studies to accelerate research focused on the development of therapeutic agents against COVID-19, which might imply further studies with promissory extracts or even screening plant species that so far do not have studies.

The anti-inflammatory potential of crude plant extracts has led to subsequent target studies for the isolation and identification of bioactive metabolites. In this sense, it has been found that most plant metabolites with anti-inflammatory activity belong to varied chemical classes, like flavonoids, polyphenols, alkaloids, terpenoids, and steroids, with some of them targeting different inflammatory pathways, which improves their therapeutic profile as multifunctional agents.

### 1.3. Polyphenols

Polyphenols are a large group of natural compounds characterized structurally by at least one aromatic ring linked to hydroxyl groups and their ubiquitous distribution in plants. These NPs comprise more than 15,000 molecules found either as aglycones or in a glycosidic form and are grouped into two major classes: flavonoids and nonflavonoids [54].

#### 1.3.1. Flavonoids

**Figure 1:**
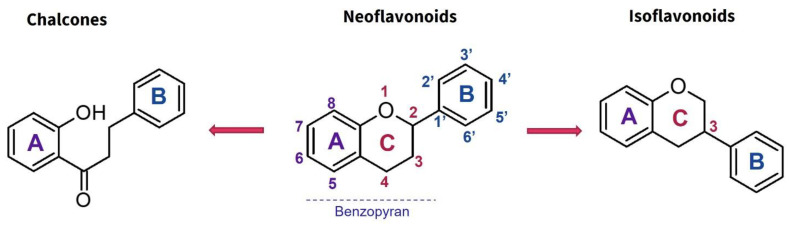
Basic scaffold of flavonoids and general classification.

Flavonoids are the most extensive group of polyphenols with a wide distribution in the plant kingdom, which has played an interesting role in their evolutionary development. These metabolites play different functions in the chemical defense and adaptive response of higher plants to biotic and abiotic stress factors, such as UV light, oxidative stress, and microbial infections [55]. At the structural level, flavonoids present a basic flavan scaffold, commonly known as C_6_-C_3_-C_6_, formed by two phenyl rings (C_6_), A and B, that are linked to a pyran (C_3_), the C ring (Figure 1). Although flavonoids have a simple skeleton compared to other polyphenols, more than 10,000 structures have been reported, which are grouped into three classes according to differences in the substitution pattern and oxidation in the C ring (Figure 1). Neoflavonoids comprise the most extensive group with six sub-classes: flavones, flavanones, flavonols, flavononols, catechins, and anthocyanins. Chalcones belong to an apart sub-class characterized by the absence of a C ring and are the biosynthetic precursors of neoflavonoids. Isoflavones have an isoflavonoid core (Figure 1) and are characterized by restricted distribution in plants since they are mainly found in the Fabaceae family [56,57].

Flavonoids are widespread metabolites in vegetables, fruits, and some beverages; hence, they are known as dietary flavonoids. In addition, these compounds have been related to beneficial properties in human health; among these, their antioxidant function is probably the most representative and extensively studied [58]. Thus, it is not surprising that flavonoids present a broad spectrum of pharmacological activities, such as neuroprotective, anti-diabetic, anti-carcinogenic, immunomodulatory, and anti-inflammatory, among others [59,60,61]. Flavonoids have shown variate molecular mechanisms in anti-inflammatory studies that allow them to control and decrease important mediators involved in inflammation. These include the inhibition of protein kinases, downregulation of transcription factors (NF-κB), inhibition of signaling transduction, and capture of reactive species (ROS, NOS) production; consequently, their activity leads a direct impact on the modulation of the immune system and inflammatory processes [62,63].

The structure, activity, results, and mechanisms reported for some promissory anti-inflammatory flavonoids, as well as possible tropical American plants, as sources of these compounds are summarized in Table 2. Undoubtedly, quercetin is not only the flavonol with the higher distribution in plants but also one of the most studied at a pharmacological level, with positive results in antioxidant, antitumoral, and anti-inflammatory activity [62]. *In vitro* and *in vivo* studies have demonstrated that quercetin acts as a multitarget ligand regulating different inflammation pathways by the activation of antioxidant genes and enzymes (Table 2). For instance, a study conducted in bone marrow-derived macrophages (BMDMs) after lipopolysaccharide (LPS 50 ng/mL) stimulation showed that quercetin reduces, in a dose-dependent manner, the secretion of TNF-α and IL-1β. Moreover, quercetin at concentrations of 1, 10, and 50 µM inhibits inducible nitric oxide synthase (iNOS) expression and downregulates the NF-κB pathway, inhibiting IkB-α phosphorylation [64,65]. In contrast to the *in vitro* and *in vivo* activity shown by quercetin, it has been found that its glycosides quercitrin and rutin (Table 2) are less active in the downregulation of the NF-κB pathway in macrophages. However, *in vivo* models showed that quercitrin (quercetin 3-rhamnoside) also inhibits the pro-inflammatory cytokine production and iNOS expression induced by dextran sulfate sodium (DSS) in rats [64]. Therefore, it is proposed that intestinal enzymes may cleave the glycosidic bond, thereby releasing the aglycone quercetin, which is capable of exerting anti-inflammatory effects [64]. On the other hand, rutin showed effective cardioprotective activity in murine models after LPS exposition, regulating cardiac marker enzymes and increasing antioxidant enzymes. In addition, this glycosylated flavonol attenuated the tumor necrosis factor α (TNF-α) and interleukin 6 (IL-6) activity [66]. Nevertheless, it is still necessary to determine the bioavailability of rutin and corroborate if glycoside is an active form or acts as a prodrug releasing quercetin, like in the quercitrin case. Finally, it is noted that the *in vivo* activity of rutin and quercitrin is a particular phenomenon because, so far, most of the anti-inflammatory reports have been made with aglycones, even though glycosylated flavonoids are widespread in natural sources. Thus, if the glycosidic forms act as prodrugs releasing aglycones there is a greater opportunity for the use of phyto-preparations (extracts and fractions) for the treatment or prevention of inflammatory conditions because glycosidic forms are found in a higher proportion in plants.

Luteolin, diosmetin, and pilloin possess a flavone core and differ in the hydroxylation degree of the A and B rings (see Table 2). Luteolin has been found to be more promissory in the modulation of immune responses compared to quercetin and other flavonoids. In fact, luteolin has been shown to decrease NF-κB transcriptional activity in mouse bone marrow-derived dendritic cells (BMDMs), peripheral macrophages, fibroblasts, and microglia after LPS activation [75]. In the BMDM model, luteolin and quercetin showed effective inhibition in TNF-α, with EC_50_ values of 10.0 µM and 20.0 µM, respectively. Also, in the same study, it was demonstrated that luteolin regulates iNOS expression, decreases NO release, and inhibits IkB-α phosphorylation at concentrations lower than 50 µM, evidencing it is an interesting flavonoid in terms of immune system modulation and inflammation [69]. Furthermore, diosmetin (a C-4′ methoxylated flavone) (see Table 2) presented lower anti-inflammatory activity in the BMDM-LPS model compared to luteolin. Indeed, diosmetin showed inhibition of TNF-α and NO production with percentages near 50% at 25 and 50 µM, while luteolin showed percentages higher than 85% at the same concentrations. Such findings suggest that hydroxylation patterns in the B ring of flavones might play a key role in the downregulation of the NF-κB pathway *in vitro*. Nevertheless, diosmetin might be considered a potential candidate for the treatment of acute pulmonary deficiency in COVID-19 patients since it has been shown to ameliorate lung inflammation in BALB/c mice after LPS stimulation, decrease pro-inflammatory cytokines, regulate the NLRP3 inflammasome, and improve tissue damage in lungs [76].

Flavanones, like pinocembrin and naringenin (Table 2), have shown beneficial properties against different chronic inflammation pathologies, such as ischemic stroke, asthma, immune disorders, and atopic dermatitis. At the molecular level, these flavonoids are able to inhibit the expression of pro-inflammatory factors and downregulation of pro-inflammatory cytokines [62]. Pinocembrin is a simple dihydroxy flavanone widely distributed in propolis and tropical plants, mainly in the Piperaceae and Asteraceae families. The different pharmacological applications of pinocembrin include neuroprotective, anti-pulmonary fibrotic, vasodilatadory, and anti-apoptotic activities [71]. Several studies indicate that pinocembrin presents anti-inflammatory activity mainly by reducing pro-inflammatory cytokines modulated by inhibition of mitogen-activated protein kinases (MAPKs) and NF-κB signaling pathways [71]. For example, in BV2 microglia cells stimulated with LPS (0.5 µg/mL), this flavonoid was able to inhibit TNF- α, IL-1β, NO, and PGE_2_ production at a concentration higher than 50 µM dose-dependently. In the same model, pinocembrin showed the regulation of iNOS and COX-2 expression and the inhibition of PI3K and Akt phosphorylation. In other *in vitro* models like RAW 264.7 cells, a down regulatory effect has been observed in TNF-α, IL-1β, and IL-6 generation after LPS and pinocembrin exposition [72]. Furthermore, the most interesting aspect of the pharmacology of pinocembrin is its pharmacokinetic profile, not only in animal models but also in humans. Preclinical and clinical studies have demonstrated that pinocembrin is easily absorbed, distributed, and metabolized, showing low bioaccumulation. Due to the huge evidence of the pharmacological potential of pinocembrin, the China Food and Drug Administration (CFDA) has approved its use for the treatment of ischemic stroke [71]. All the above makes this flavonoid a potential candidate for the treatment of chronic inflammatory conditions; consequently, it would be interesting to determine its potential in models related to infectious diseases such as COVID-19.

Other dietary flavonoids like naringenin (Table 2) might have therapeutic properties against COVID-19 due to their antiviral, immunomodulatory, and anti-inflammatory activities [73]. The immunomodulatory and anti-inflammatory properties of naringenin have even been studied in models related to respiratory syndromes. As an example, the immunomodulatory mechanisms triggered by naringenin in macrophages include the regulation of key signaling pathways such as NF-κB and MAPK [77]. Also, in acute respiratory distress syndrome *in vivo*, naringenin is shown to decrease lung injury by reducing oxidative stress and diminishing neutrophils [73,77]. Therefore, considering the key link between neutrophil overactivation and the cytokine storm in COVID-19, as well as the key role of NF-κB, naringenin represents an interesting natural option for the treatment of patients with comorbidities or severe cases of SARS-CoV-2 infection.

Catechins like epicatechin and epigallocatechin-3-gallate (EGCG) are the main constituents of green tea (*Camelia sinensis*). Structurally, they are characterized by the absence of the carbonyl group on the C ring (Table 2) and present anti-inflammatory properties. EGCC has shown the inhibition of Myeloperoxidase (MPO) and the competitive inactivation of pro-inflammatory chemokines (CXCL9, CXCL10, and CXCL11), which leads to regulation of the hyperinflammatory response in macrophages and neutrophils [78]. In addition, EGCG was able to diminish the expression of TNF α, IL 1β, IL 6, and IL 8 in different cell models after LPS stimulation, suggesting its pharmacological potential on inflammatory pathologies [74]. However, it is important to emphasize that catechins have shown lower multitarget anti-inflammatory activity compared to flavones and flavonols.

**Figure 2:**
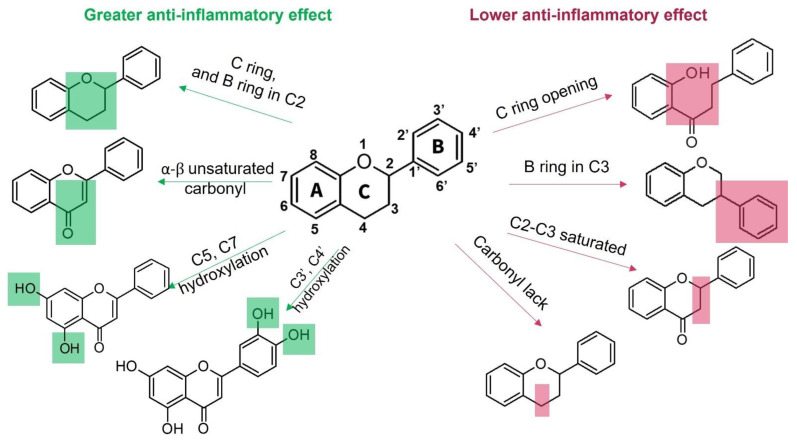
Structural motifs associated with anti-inflammatory effects in flavonoids.

Moreover, flavonoids, such as isoflavones and chalcones, which present modifications in the benzopyrone scaffold (Figure 1), have not shown marked anti-inflammatory and immunomodulatory properties compared to neoflavonoids [63]. Thus, some structural features have been suggested to be associated with anti-inflammatory activity in flavonoids; among these, the presence of heterocycle C, oxidation degree of the C ring, and hydroxylation patterns in the A and B rings stand out (Figure 2). Some studies support that flavonoids with a B ring in the C2 position are more likely to attenuate different signaling pathways involved in inflammatory processes, which would explain why neoflavonoids present higher activity than isoflavones [69]. In addition, unsaturation at C2–C3, conjugated with the carbonyl in C4 (ring C), is believed to increase the anti-inflammatory effects in the flavonoids on the basis that flavones and flavanols show the highest activity. By contrast, flavanones, flavanonols, and catechins, which lack at least one of the above structural conditions, are usually less active [79]. Finally, it has been suggested that meta-hydroxylation patterns at the A ring (C5 and C7) and ortho-hydroxylation in the B ring (C3′ and C4′) improve not only the antioxidant activity but also the modulation of enzymes and transcription factors involved in inflammatory pathologies (Figure 2).

Therefore, regarding the above reports, it is possible to deduce that flavonoids like luteolin, apigenin, and quercetin might be a good starting point in the development of alternative phytotherapy for acute COVID-19 infections. These natural flavonoids have a wide distribution in plant species in the tropics of America, mainly as glycosides or aglycones in edible plants. In addition, previous studies have shown that these polyphenols present multitarget characteristics in pathways related to hyperinflammation and immune response, mainly by the downregulation of nuclear factor (NF)-κB, which plays a key role in the cytokine storm. Finally, free flavonoids and their glycosides have shown an excellent pharmacokinetic profile in animals and humans, which enhances their drug-likeness profile.

#### 1.3.2. Non-Flavonoid Polyphenols

By contrast to flavonoids, non-flavonoid polyphenols (NFPs) do not share a common scaffold; in fact, they present varied structures grouped in sub-classes, such as stilbenes, lignans, tannins, phenylpropanoid acids, and hydroxybenzoic acids. Nonetheless, like flavonoids, NFPs are also present in edible plants; indeed, about 30% of the total dietary polyphenols correspond to phenolic acids [54]. Moreover, these polyphenols have shown different pharmacological activities related to their antioxidant properties. Due to the positive health effects associated with their consumption, polyphenols are highly promising in drug development or preventive therapy [80].

Certainly, curcumin and resveratrol (Table 3) are the two most popular polyphenols due to their endless reports of biological and pharmacological activities. Also, both have been obtained from plants mostly distributed in tropical regions of the world [81]. Resveratrol and other stilbenes are the majority polyphenols in grapes, blackberries, and teas, whereas curcumin and other curcuminoids are restricted metabolites of the *Curcuma* genus [82,83]. At the pharmacological level, these compounds are characterized by exerting a broad range of activities that have been associated with their antioxidant and anti-inflammatory capacities. More than 2000 research papers related to the anti-inflammatory activity of these polyphenols have been published in the past decade. Even more, the studies of the molecular mechanisms involved in their anti-inflammatory activity suggest that these polyphenols are multitarget ligands, modulating different key signaling pathways [82,83]. Nevertheless, recently, controversy has been sparked about the scaffold promiscuity rather than the real polypharmacological effect of these small molecules. As a matter of fact, some molecular docking and dynamic studies have shown that resveratrol displays similar binding modes and target interactions with various proteins; thus, special precaution is required concerning the possible off-target effects [84]. However, it is certain that a large number of *in vitro* and *in vivo* studies have shown the promissory pharmacological potential of resveratrol, but so far, there is no real evidence of its effects on human health from clinical studies [83].

On the other hand, hundreds of clinical trials support the beneficial effects of curcumin for the treatment of inflammatory conditions; hence, varied pharmaceutical forms of curcumin and other curcuminoids have been approved by the FDA [88] (Hewlings and Kalman 2017). Curcuminoids are a particular group of natural polyphenols that share a diarylheptanoid core and are mainly found in the *Curcuma* genus. Particularly, curcumin (Table 3) is the major polyphenol of *Curcuma longa*; as mentioned above, it is one of the most ancient species in Asian folk medicine. Curcuminoids have been shown to be the active principles of turmeric, as they exert anti-inflammatory and antioxidant activity, both *in vitro* and *in vivo*. In fact, curcumin and its analogues attenuate several inflammatory responses by multitarget mechanisms, including downregulation of the NF-κB signaling pathway [82]. Consequently, these polyphenols might be capable of diminishing the cytokine storm involved in acute lung infection caused by SARS-CoV-2. Moreover, several studies have shown the antiviral potential of curcumin against viruses that affect the respiratory system, including influenza and respiratory syncytial virus, which could expand their therapeutic potential to COVID-19 [89]. In spite of its anti-inflammatory and antioxidant effects, curcumin has a poor pharmacokinetic profile, which is the main drawback at the pharmacological level. This presents as poor absorption, fast metabolism, and elimination, which leads a deficient bioavailability, sometimes with undetectable concentrations after administration. In this context, different strategies have been designed to overcome this limitation. Among them, the most successful is the co-administration of curcumin and Piperine (the major bioactive of black pepper), which allows the bioavailability of this polyphenol to be enhanced by about 2000% [88]. Moreover, this combination of NPs could display synergistic anti-inflammatory properties considering the activity of Piperine, which would be an excellent and accessible option for the treatment of infectious diseases like those caused by viruses [90].

Phenylpropanoid (C_6_C_3_) and hydroxybenzoic (C_6_C_1_) acids are biosynthetic building blocks of more complex NPs, such as lignans, flavonoids, tannins, and even alkaloids, and due to their biosynthetic role, they are commonly widespread in plants [91]. Hydroxycinnamic acids, such as ferulic acid, are characteristic members of this group of polyphenols and are characterized by their antioxidant and anti-inflammatory action (Table 3). For example, both showed anti-inflammatory activity in macrophage models, reducing the production of pro-inflammatory cytokines after LPS exposure [24]. As a result of their promising activity, these compounds have served as the basis for the design of hybrid ligands in order to enhance the anti-inflammatory effect as well as pharmacokinetic properties [85]. Moreover, other natural derivatives of caffeic acid, such as Rosmarinic acid, present high anti-inflammatory potential (Table 3). Rosmarinic acid could be considered one of the most important polyphenols in the search for therapeutic options for complex inflammatory diseases because it can be obtained from different tropical plants with high yields. This polyphenol was first isolated from *Rosmarinus officinalis* (rosemary); however, it has also been isolated from other Lamiaceae species, mainly in the *Salvia*, *Mentha*, and *Occinmun* genera, which are recognized in folk medicine for being used for the treatment of inflammatory and pulmonary conditions [87]. Also, dozens of biological activity studies of plants with a high content of Rosmarinic acid have confirmed its pharmacological potential as an anti-inflammatory and antiviral [87]. For example, Rosmarinic acid showed promising results in lung injury induced by LPS in mice since it was able to reduce the production of the pro-inflammatory cytokines TNF-α, IL-6, and IL-1b by the downregulation of the ERK/MAPK signaling pathway. As a result, a minimization in tissue damage was observed [86]. Moreover, other *in vivo* studies suggest that Rosmarinic acid regulated the production of pro-inflammatory cytokines induced after infection with influenza virus in mice, decreased oxidative stress biomarkers, and showed a pleiotropic effect on viral pneumonia [86]. The evidence above opens a gateway for the use of this polyphenol in the treatment of infectious diseases such as COVID-19.

### 1.4. Alkaloids

Alkaloids constitute one of the widest classes of natural products found in marine and terrestrial organisms and whose main characteristic is their nitrogen-containing base scaffold. In the plant kingdom, alkaloids have been mainly obtained from angiosperms, playing a crucial role in chemical defense as an adaptation and evolutionary strategy [92]. This ecological trait could be related to their huge structural diversity. It is estimated that there are more than 12,000 different natural alkaloids, which comprise simple scaffolds to complex structures, such as vinblastine. Despite their extensive chemical landscape, alkaloids are biosynthesized from small building blocks, mainly amino acids. In addition, these metabolites have been found to possess various pharmacological activities, such as anticancerogenic, antimicrobial, analgesic, and anti-inflammatory [93].

In spite of alkaloids’ structural diversity, only a few classes have shown anti-inflammatory activity, which suggests that these selected groups hold the necessary structural features to modulate targets related to inflammation. The majority of anti-inflammatory alkaloids belong to the five major classes indole, carbazole, β-carboline, quinoline, and isoquinoline, classified according to the biosynthetic pathway and the precursor amino acids [94]. These alkaloids have shown anti-inflammatory effects with multiple molecular mechanisms. Consequently, they might be used in the treatment of different inflammatory conditions. Table 4 presents selected examples of alkaloids that have shown anti-inflammatory activity by the modulation of biochemical processes during the cytokine storm.

Indole, carbazole, and β-carboline alkaloids are biosynthesized from tryptophan or tryptamine. Structurally, they share a base scaffold consisting of a pyrrole attached to a benzene ring. Carbazoles and carbolines usually present extended aromatic systems with other benzene rings or aromatic heterocycles. These tryptophan-derived alkaloids have mainly shown effects on the central nervous system (CNS); however, some of them also present anti-inflammatory activity [94]. For example, indole alkaloids isolated from species belonging to the *Uncaria* genus (Rubiacea), such as strictosidine (Table 4), have shown potent inhibitory activity of NO production in cellular models after stimulation with LPS, with IC_50_ values in the micromolar range [95]. Likewise, mitraphylline isolated from the chloroformic bark extract of *Uncaria tomentosa* exhibited anti-inflammatory activity in murine models by the regulation of pro-inflammatory cytokines (Table 4). These findings agree with the ethnobotanical use of *Uncaria tomentosa*, commonly known as “*Uña de Gato*” in South America, where the decoction is traditionally used to treat inflammatory conditions [96]. Thus, considering the traditional uses and high diversity of the *Uncaria* genus in the tropical region of America, it constitutes an interesting source of anti-inflammatory alkaloids, which may have application in the development of new therapeutic agents for emerging and re-emergent infectious diseases.

On the other hand, carbazole or dibenzopyrrole alkaloids widely distributed in the Rutaceae family, particularly in the *Murraya* and *Clausena* genera, which are native to tropical regions, and present the highest number of anti-inflammatory reports [97]. For instance, 15 carbazole alkaloids were isolated from *Murraya koenigii*, including mukolidine and O-methylmurrayamine, which showed *in vivo* and *in vitro* anti-inflammatory activity in a varied type of assay [94]. Both alkaloids were able to inhibit the production of pro-inflammatory cytokines, specifically TNF-α and IL-6, in a dose-dependent manner, with micromolar IC_50_ values that are comparable with quercetin [97]. It should be noted that, even though *Murraya* species are found abundantly in the American tropics, most of the anti-inflammatory studies have been carried out with Asian plants, which opens the opportunity to explore the chemical compositions and bioactivity of *Murraya* species from South and Central America that have not been studied so far. Lastly, β-carboline alkaloids characterized by the presence of a pyridine ring fused with the indole scaffold have also displayed downregulation in the production of pro-inflammatory cytokines [94]. As an example, harmine (Table 4), a β-carboline isolated from the *Peganum* species, showed the regulation of NF-κβ transcription and diminished pro-inflammatory cytokines, such as TNF-α, IL-1β, and IL-6, both *in vivo* and *in vitro* [94]. Also, in the mice–LPS model, harmine was able to ameliorate lung injury. Although β-carboline and carbazole alkaloids possess activity against immunological factors involved in inflammatory diseases, further toxicity studies are needed in view of its planar structure, which has been related to DNA methylation and high toxicity [100].

Isoquinolines are a large family of alkaloids biosynthetically obtained from the aromatic amino acids phenylalanine and tyrosine. In plants, isoquinolines have an ancient phylogenetic distribution in basal angiosperms, specifically in the Berberidaceae, Papaveraceae, Annonaceae, and Rutaceae families [101]. Due to their structural diversity, isoquinolines are subdivided into 13 subgroups. Among the most common are aporphines, benzophenanthridines, bisbenzylisoquinolines, phthalides, morphinan, and protoberberines [102]. At a pharmacological level, these alkaloids are characterized by their antitumor, antimicrobial, anti-inflammatory, and analgesic activities [103,104]. Berberine is the most common protoberberine alkaloid that has been isolated from different tropical species, mostly in the *Berberis*, *Coptis*, and *Zanthoxylum* genera [105]. This isoquinoline alkaloid is perhaps one of the most studied due to its polypharmacological effects and has shown antitumor, neuroprotective, anti-inflammatory, and antidiabetic activity [106]. In regard to antidiabetic properties, berberine promoted glucose and lipid metabolism in clinical trials; therefore, different formulations have been approved as a supplementary treatment for diabetes type II [107]. Recently, pharmacological studies of berberine have focused on its anti-inflammatory and immunomodulatory effects, demonstrating its multimodal activity. This alkaloid can regulate or even block the inflammatory response by ceasing the production of pro-inflammatory cytokines (TNF-α and IL-6) and PEG-2, as well as regulating the expression of COX-2, MMP-2, and MMP-9 through the downregulation of NF-κB and MAPK signaling pathways [98]. In addition, berberine can inhibit or regulate the production of pro-inflammatory cytokines, such as IFNγ, IL-6, IL-1β, IL-2, IL-17, and IL-22 in different immune–inflammatory models, which leads to the inhibition in the differentiation of pro-inflammatory Th1 and Th17 cells [108]. Given the anti-inflammatory and immunomodulatory properties of berberine, along with its additional effects on diabetes modulation—a key comorbidity in COVID-19 patients—it would be of interest to investigate the potential effects of this alkaloid in models of the disease.

Furthermore, glaucine (Table 4) and oxoglaucine, two aporphine alkaloids isolated from yellow hornpoppy (*Glaucium flavum*), have demonstrated anti-inflammatory potential by reducing the production of NO and pro-inflammatory cytokines in macrophages after stimulation with different Toll-like receptor–ligands (Table 4) [99]. Both chemicals were shown to inhibit the production of TNF-α and IL-6 in murine macrophages after stimulation with LPS (1 μg/mL). In addition, under zymosan activation, these compounds are shown to increase the production of IL-10, an anti-inflammatory cytokine involved in pathogen elimination that has also been indicated as a biomarker in patients with acute cases of COVID-19, who present low levels of this cytokine. These results suggest that glaucine and oxoglaucine could not only show anti-inflammatory activity but also favor the control of pathogen infections [99].

On the other hand, benzophenanthridines, such as nitidine, norchelerythrine, and nornitidine (Table 4), extensively distributed in *Zanthoxylum* species have demonstrated analgesic and anti-inflammatory activity through regulation of the NF-κB signaling pathway, decreasing iNOS expression and TNF-α, IL-1b, and IL-6 production [109]. Phenethylisoquinolines are an unusual sub-class of isoquinoline alkaloids principally distributed in the Liliaceae family. Among them, tropolones, like colchicine (Table 4), are characterized by an amino acid as a precursor and the presence of a non-heterocyclic N (protoalkaloid). Colchicine was originally obtained from *Colchicum autumnale,* an ancient plant recognized for its pain-release and anti-inflammatory properties. Even though this alkaloid has been recognized as one of the oldest natural anti-inflammatories, its use was only officially approved by the FDA in 2009 [110]. Recently, there has been growing interest in the potential use of colchicine for the treatment of acute cases of COVID-19 due to its anti-inflammatory effects [28]. Several studies indicate that colchicine inhibits the activation of NLRP3 inflammasome, leading to a potent reduction of IL-1β production, which, in turn, prevents the cytokine storm. Additionally, preclinical studies and clinical trials support the possible effects of colchicine both in the initial phase and progression of the inflammatory process in COVID-19; however, dosage, safety, and toxicity studies are still required [28].

**Figure 3:**
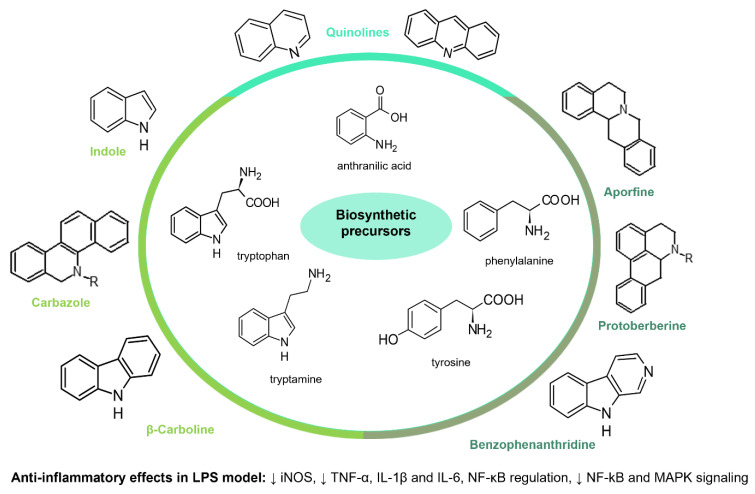
Frequent scaffolds in anti-inflammatory alkaloids and their biosynthetic precursors.

The huge chemical diversity of alkaloids has hindered structure–activity relationship (SAR) studies in figuring out the pharmacophoric motifs linked to the anti-inflammatory effects of alkaloids. However, it is worth pointing out that a high percentage of the anti-inflammatory alkaloids reported so far, share a common chemical feature: biosynthetic precursors (Figure 3).

As mentioned above, indole, carbazole, β-carboline, quinoline, and isoquinoline are obtained directly from aromatic amino acids (tryptophan, phenylalanine, and tyrosine) or from derived precursors (anthranilic acid o tryptamine), most of them have shown related anti-inflammatory activity by modulating some key signaling pathways (Figure 3). The foregoing could be a clue in drug design for the development of anti-inflammatory therapeutic agents, not only for COVID-19 but also for infectious diseases in general.

### 1.5. Terpenoids and Saponins

Terpenoids are a vast and structurally diverse class of naturally occurring compounds, mostly with lipophilic character and biosynthetically obtained from a common precursor through the mevalonate pathway. These NPs are also known as isoprenoids because their carbon skeletons are formed by C_5_ units, whose common building block is the isoprene unit. For this reason, terpenoids are grouped in different classes accordingly to the C5 units: hemiterpenes (C5), monoterpenes (C10), sesquiterpenes (C15), diterpenes (C20), sesterpenes (C25), triterpenes (C30), and tetraterpenes (C40). In plants, terpenoids fulfill key survival functions, which it is linked to their ubiquity, for example, sterols and triterpenes are the main components of cell membranes, while monoterpenes and sesquiterpenes play important roles in chemical ecology, as attractants for pollinators, or defense against pathogens [111]. Related to their ecological functions, terpenoids have presented different biological activities such as antimicrobial, antitumor, hepatoprotective, and anti-inflammatory [111]. Some terpenoids, mostly sesquiterpenes, diterpenes, and triterpenes, have shown anti-inflammatory potential by regulation of cytokine secretion and modulation of key signaling pathways involved in the infection-triggered inflammatory response [112].

Monoterpenoids are the major components of several essential oils obtained from aromatic plants, some of them have been traditionally used in the treatment of inflammatory and/or respiratory conditions. Hence, different studies have been focused on the anti-inflammatory activity of essential oils and their major constituents to corroborate their uses in folk medicine [113]. Such is the case of 1,8-cineole (Table 5), also known as eucalyptol, that constitutes 90% of the eucalyptus essential oil; this monoterpenoid have shown anti-inflammatory activity both *in vivo* and *in vitro* models [114]. In human monocytes after stimulation with LPS, eucalyptol exhibited to inhibit the TNF-α production, as well as, regulation of pro-inflammatory cytokines and other inflammatory molecules such as leukotriene (LTB_4_) and thromboxane (TxB_2_) [114]. Complementary studies have demonstrated that the inhibition of LTB_4_ and TxB_2_ production is linked to the disruption in the arachidonic acid (AA) metabolism pathway. Moreover, eucalyptol has shown strong inhibition of pro-inflammatory cytokines in human monocytes and lymphocytes after LPS exposition; indeed, at a concentration of 1.5 µg/mL of this terpene, a reduction higher of 80% was obtained for TNF-α and IL-1β in both cellular models [114]. Thus, considering the important role of those mediators in acute inflammatory responses, the anti-inflammatory capacity of eucalyptol has also been investigated in guinea pig models, detecting a significant reduction of these pro-inflammatory cytokines in bronchoalveolar fluids [114]. Lastly, clinical studies with 1,8-cineole have also been carried out in patients with chronic obstructive pulmonary disease, a significant improvement in pulmonary function and reduction of airway inflammatory response were observed after administration of 200 mg of cineole three times per day for six months [113]. Other monoterpenes highly distributed in essential oils such as menthol, borneol, carvacrol, terpinene-4-ol, pulegone, geraniol, and menthone have shown anti-inflammatory potential with similar effects to 1,8-cineole [113], which hinted at the pharmacological potential of essential oils for the treatment of infectious respiratory diseases. Furthermore, these scientific findings support the traditional uses of different aromatic plants used for the treatment of respiratory diseases caused by viruses or bacteria, associated with acute inflammatory responses.

Sesquiterpenoids (C15) are the most diverse group of terpenoids, comprising linear, monocyclic, bicyclic, and tricyclic structures; for this reason, they are clustered in several sub-classes regarding the carbonate backbone. These terpenoids have been isolated from essential oils and nonpolar fractions in aromatic and medicinal plants, being profuse in the Asteraceae family. In the pharmacological field, sesquiterpenoids have demonstrated antitumoral and anti-inflammatory potentiality, in many cases with activities related to their plant sources [115]. The sesquiterpenoids isolated from various species of *Curcuma* genus have shown an anti-inflammatory capacity even greater than that of polyphenols, which traditionally have been considered the active constituents of these species [82]. Curcumol (Table 5) a hemiacetal guaiane-type sesquiterpenoid found in the essential oils of rhizomes in the *Curcuma* genus species displayed substantial anti-inflammatory activity by suppressing JNK- mediated AP-1 signaling pathway in RAW 264.7 cells after stimulation with LPS. In this *in vitro* model, curcumol showed inhibition of NO production by restraining the iNOS expression, as well as, reduction of TNF-α, IL-1β, and IL-6 production [116]. Currently, different presentations of phytotherapeutics with curcumol are sold for the treatment and prevention of inflammatory disorders and cancer.

**Table 5:** Selected examples of terpenoids and saponins, structures, anti-inflammatory effects, and potential sources in tropical plants.

Compound (Sub-Class)	Structural Formula	Model/Assay	Effects and Mechanism	Sources in Tropical Plants	Ref.
1,8-cineole (monoterpenoid)	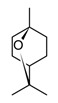	*In vitro* Human monocytes-LPS *In vivo* Guinea pigs	↓ TNF-α production ↓ IL-1β, LTB_4_, TxB_2_ ↓ TNF-α and IL-1β	*Eucalyptus* spp. *Salvia* sp. *Cinnamomun* *Laurus nobilis*	[113,114]
Geraniol (monoterpenoid)	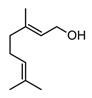	*In vitro* RAW 264.7-LPS	↓ NO, PGE2 ↓ iNOS and COX-2 expression ↓ NF-κB	*Pelargonium* spp. *Citrus* oils *Cymbopogon* spp.	[113]
Curcumol (sesquiterpenoid)	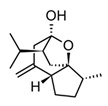	*In vitro* RAW 264.7-LPS *In vivo* Mice-LPS	↓ iNOS expression ↓ NO production ↓ TNF-α, IL-1β and IL-6 ↓ regulation JNK-AP-1	*Curcuma* essential oils	[116]
Parthenolide (sesquiterpenoid)	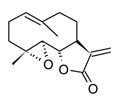	*In vitro* PBMCs-LPS *In vivo* Balb/c female mice-LPS	↓ iNOS and COX-2 expression ↓ TNF-α, IL-1, IL-4, IL-8 and IL-12 ↓ NF-κB	*Tanacetum* spp.	[117]
Epi-eudebeiolide C (sesquiterpenoid)	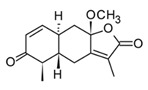	*In vitro* RAW 264.7-LPS	↓ iNOS expression ↓ NF-κB ↓ IkB phosphorylation	*Salvia plebeia*	[115]
Andalusol (diterpenoid)	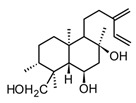	*In vitro* J744 cells-LPS	↓ iNOS expression ↓ NF-κB	*Siderits foetens*	[112]
Salofficinoid G (diterpenoid)	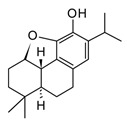	*In vitro* RAW 264.7-LPS	↓ iNOS and COX-2 expression ↓ NO	*Salvia* *officinalis*	[118]
Lupeol (triterpenoid)	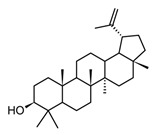	*In vitro* Macrophages	Block Akt pathways ↓ NF-κB ↓ PGE2 production	*Mangifera indica*, *Zanthoxylum* spp. *Tamarindus* spp. *Celastrus* spp.	[119]
Ursolic acid (triterpenoid)	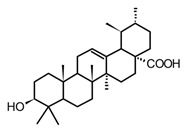	*In vitro* RAW 264.7-LPS	↓ iNOS and COX-2 expression ↓ PGE2 production ↓MAPK, TNF-α, IL-6, IL-1β, TLR4	*Ocimum sanctum* *Thymus vulgaris* *Lavandula* spp. *Nepeta sibthorpii* *Mentha piperita*	[119]
Diosgenin (steroid from the saponin Dioscin)	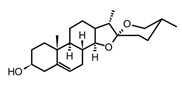	*In vitro* Macrophages-LPS	↓ ICAM-1 expression ↓ NF-κB ↓ MAPK, Akt, IKKβ	*Solanum* spp. *Dioscorea* spp.	[119]

Abbreviations: Akt, Serine/threonine protein kinase Akt; ICAM-1, Intercellular adhesion molecule-1; IKKβ, Inhibitor of nuclear factor kappa-β kinase subunit beta; IL, interleukin; LTB_4_, leukotriene B4, LPS, lipopolysaccharide; MAPK, mitogen-activated protein kinase; NO, nitric oxide; NOS, nitric oxide synthase; NF-κB, nuclear factor–kappa β; PBMCs, peripheral blood mononuclear cells; PGE_2_, prostaglandin E_2_; SOD, superoxide dismutase; TNF, tumor necrosis factor; TxB2, tromboxane B2. Cell line: J744 cells, murine macrophages, derived from a BALB/c mouse tumor. The value (expression or concentration) decreased (↓).

Among sesquiterpenoids, the sesquiterpene lactones present more restricted distribution in plants, they are characterized by a γ-lactone system fused to the sesquiterpene core sub-divided into three major classes guainolides, germacranolides, and eudesmanolides. Sesquiterpene lactones isolated from the Asteraceae family comprise the highest percentage of the total reported so far, these metabolites have shown biological and pharmacological significance, within is possible to underline its marked anti-inflammatory and anti-cancer activities [120]. Surely, the most relevant example of those bioactive metabolites is parthenolide (Table 5), a germacranolide-type sesquiterpene lactone present in several anti-inflammatory plants. Parthenolide was initially isolated from the medicinal plant *Tanacetum parthenium* (Asteraceae), also popularly known as Feverfew, used to treat fever, headache, and some inflammatory problems [121]. Parthenolide has shown anti-inflammatory effects by multiple molecular mechanisms, which makes it a potent multitarget agent against inflammatory disorders. Among the mechanism related to cytokine storm, this sesquiterpene lactone can inhibit the expression of nitric oxide synthase (iNOS) and pro-inflammatory cytokines such as TNF-α, IL-1, IL-4, IL-8, and IL-12 [122]. *In vitro* and *in vivo* studies have demonstrated that parthenolide exerts anti-immune–inflammatory activity downregulating the transcription factor NF-κB through the direct union to the IκB kinase complex (IKK) which hindering the IkB phosphorylation and leads to the deactivation of the transcription factor [121]. In addition, this compound can inhibit the NLRP3 inflammasome, independently of the regulation of the NF-κB pathway [117]. Thus, considering the role of inflammasomes in the detection of external pathogens and activation of pro-inflammatory cytokines through caspase 1, this trait could have a synergistic effect in mediating the hyperinflammatory response in acute cases of infectious diseases. Regardless of the potent anticancer and anti-inflammatory activity of parthenolide, the pharmacokinetic characteristics, low water solubility, and poor bioavailability limit its pharmacological applications. Therefore, new strategies are needed to improve the pharmacokinetic profile of sesquiterpene lactones and expand its applications in drug development.

Diterpenoids (C20) are less abundant compared to mono and sesquiterpenes, these terpenoids have been found in higher plants, fungi, and marine organisms; being marine diterpenes one of the most valuable sources in terms of scaffold diversity. In plants, diterpenoids have a slightly restricted distribution and have been isolated from Asteraceae and Lamiaceae families [118]. Similarly, to sesquiterpenes, they are characterized by their significant anticancer and anti-inflammatory activity. Most of the diterpenoids exert their anti-inflammatory activity by inhibiting or downregulating the NF-κB signaling pathway; some labdane-type diterpenes obtained from Lamiaceae species like andalusol (Table 5) have shown inhibition of the NOS expression and the phosphorylation of IkB in murine macrophages [112]. *S. officinalis* (Lamiaceae) is an aromatic herb cultivated in tropical regions worldwide traditionally used in folk medicine as antiseptic and anti-inflammatory. Chemical studies have been associated with some norabietane diterpenoids with the anti-inflammatory activity of this medicinal plant [118]. The *in vitro* study using RAW 264.7 murine macrophages of twelve diterpenoids isolated from *S. officinalis* showed that salofficinoid G (Table 5) can inhibit the NO production in a dose-dependent manner. Also, this compound showed anti-inflammatory effects blocking the expression of inflammatory enzymes COX-2 and iNOS and downregulating the mitogen-activated protein kinase (MAPK) signaling pathway after stimulation with LPS [118].

Triterpenoids (C30) share the main scaffold composed of five or six rings, usually with different degrees of oxygenation or substitution. The patterns in the rings assembly and substitutions give rise to 20 different sub-classes, among the most common: lanostanes, dammaranes, lupanes, oleananes, and ursanes. In addition, as an adaptive strategy some plants store triterpenoids in form of saponins, a special class of triterpenoid glycosides highly polar and moderately toxic. Through their physiological functions, triterpenoids are not only a huge group of secondary metabolites with almost ubiquitous distribution in plants, they have shown a wide range of pharmacological effects [123]. It is well known that triterpenoids are important constituents of the cell membrane in plants, stabilizing the phospholipidic bilayers, while some of them play key hormonal roles in plants, those structural features have been correlated to their anti-inflammatory properties. Within the large group of triterpenoids that have shown anti-inflammatory effects, Jeong and Bae [119] selected the 12 most promising ones, among these lupeol, ursolic acid, and diosgenin (Table 5) have been isolated from different species present in the American tropical region. These two triterpenes and saponin showed anti-inflammatory activity both in *in vitro* and *in vivo* tests modulating NF-κB signaling pathway by one or more related mechanisms [119].

In general, based upon this evidence it is possible to conclude that a high percentage of terpenoids exerts their anti-inflammatory activity through the regulation of the NF-κB signaling pathway. Thus, considering that this transcription factor is activated by Toll-like receptor ligands triggering a cascade of pro-inflammatory molecules such as cytokines (IL-1β, IL-6) those natural compounds might play a favorable role in the control of inflammatory response caused by infectious agents such as viruses. In addition, at the sight of the promising anti-inflammatory activity of terpenoids obtained from species of the Asteraceae and Labiaceae families, it is important to point out, that although the tropic region of America has the greatest diversity of Asteraceae and Lamiaceae plant species almost 80% of the studies reported in this area belong to species from the tropical and subtropical regions of Asia. Therefore, there is still a great chemical and biological potential unexplored in the tropical American region, and it is our duty to contribute to the search for bioactive molecules that can be a starting point in the control of the current pandemic and those that might be in the future.

## 2. Conclusions

At the sight of the current pandemic and future infection outbreaks that threaten the economy and global public health, it is necessary to explore different options for the development of more effective and safe therapeutic agents to save thousands of lives. In this sense, plants, which have been used for thousands of years to treat different ailments, are one of the most impressive sources of small molecules with privileged scaffolds. Moreover, countless scientific studies of medicinal plants have shown that popular knowledge constitutes a cornerstone in the search for bioactive natural products-plant derived. To date, several plant metabolites such as polyphenols, alkaloids and terpenoids have been shown to have anti-inflammatory activities by modulation of pathways involved in cytokine storms.

## 3. Perspectives

Considering the role of the hyperinflammatory cascade in the progression and severity of COVID-19, some of these metabolites could play a crucial function as a possible therapeutic strategy for this disease. Natural products such as luteolin, quercetin, curcumin, colchicine, 1,8-cineole and parthenolide, among others, have shown important anti-inflammatory and immunomodulatory properties by regulating key signaling pathways associated with SARS-CoV-2 infection. However, further preclinical, or clinical studies are still required to determine the real potential of these metabolites for the treatment of severe cases of cytokine storms. On the other hand, it would be worth undertaking studies of medicinal plants in the tropic region of America that have not been explored so far, in order to have alternative sources of novel small molecules that not only contribute at chemical level but also in the pharmacological knowledge of them.

## Figures and Tables

**Table 1:** Selected tropical American plants with potential anti-inflammatory activity.

Family	Botanical Name	Part Used	Extract	Model/Assay	Results	Ref.
Anacardiaceae	*Anacardium occidentale* L.	Leaves	Ethanol/water (40:60 *v*/*v*)	RAW 264.7-LPS	↓ TNF-α, IL-1β, and IL6 ↓ Inducible NO and ROS	[43]
	*Mangifera indica*	Stem, bark	Aqueous	RAW 264.7-LPS/IFN-γ and calcium ionophore A23187	↓ TNF-α Inhibition of PGE_2_ and LTB_4_ (IC_50_ < 25µM)	[45]
Asteraceae	*Achyrocline satureioides*	Aerial parts	Aqueous	Human PMNs and PBMCs	Regulation of IFN-γ/IL-4 ratio ↓ ROS production	[46]
	*Baccharis dracunculifolia*	Leaves	Ethanol/water (70:30 *v*/*v*)	Murine macrophages–LPS	NF-κB downregulation ↓ IL-1β, IL-6, and IL-10 ↓ ROS production	[43]
Fabaceeae	*Caesalpinia ferrea*	Fruits (pods)	Aqueous	Different *in vitro* and *in vivo*	↓ TNF-α, IL-1β, NO, and TGF-β	[43]
	*Copaifera multijuga*		Oleoresin	Murine macrophages	↓ TNF-α, IL-1, and IL-6 Inhibition of NO release	[43]
Lamiaceae	*Hyptis pectinata*	Leaves	Essential oil	Balb/C mice	↓ TNF-α, PGE_2_, and IL-6	[43]
Lauraceae	*Persea americana* Mill	Leaves	Ethanol/water (1:1)	RAW 264.7-LPS	↓ TNF-α gene expression Inhibition of NO release	[47]
Petiveriaceae	*Petiveria alliacea*	Leaves	Ethanol	RAW 264.7-LPS	NF-κB downregulation ↓ PGE_2_, iNOS, and NO ↓ IL-1β, IL-6, and IL-10	[48]
Plantaginaceae	*Scoparia dulcis*	Whole plant	Ethanol/water (70:30 *v*/*v*)	ICR mice	↓ COX-2, NO, TNF-α, and IL-1β	[43]
Rubiaceae	*Uncaria tomentosa*	Root, bark	Aqueous	RAW 264.7-LPS	NF-κB downregulation ↓ IL-1, IL-17, and TNF-α	[43]
Solanaceae	*Physalis angulata*	Calyx	DCM	RAW 264.7-LPS ICR mice–SOZ	↓ PGE_2_ and NO production ↓ IL-1β, IL-6, and TNF-α	[49]

Abbreviations: DCM, dichloromethane; IC_50,_ inhibitory concentration 50; ICR mice, outbred albino mice from the Institute of Cancer Research; IL, interleukin; LPS, lipopolysaccharide; NF-κB, nuclear factor–kappa β; NO, nitric oxide; NOS, nitric oxide synthase; PBMCs, peripheral blood mononuclear cells; PGE_2_, prostaglandin E_2_; PMNs, polymorphonuclear leukocytes; ROS, reactive oxygen species; SOD, superoxide dismutase; SOZ, serum-opsonized zymosan; TNF, tumor necrosis factor. Cell line: RAW 264.7, murine macrophages originally derived from BALB/c mice. The value (expression or concentration) decreased (↓).

**Table 2:** Selected examples of flavonoids, their anti-inflammatory effects, and sources in tropical plants.

Compound (Sub-Class)	Structural Formula	Model/Assay	Effects and Mechanism	Source in Tropical Plants	Ref.
Quercetin (flavonol)	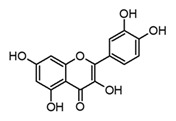	*In vitro* BMDM-LPS	↓ TNF-α and IL-1β ↓ iNOS expression ↓ IkB-α phosphorylation	*Carica papaya* *Anacardium occidentale* *Capsicum annum* *Momordica charantia* *Moringa oleirfera* *Psidium guajava* *Amaranthus* spp. *Curcuma* spp.	[64,67]
Quercitrin (flavonol glicoside)	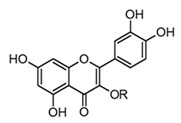 R = α-l-rhamnopyranoside	*In vivo* Rats–DSS	↓ TNF-α and IL-1β ↓ iNOS expression	*Allamanda cathartica* *Euphorbia* spp. *Myrtus* spp.	[64,68]
Rutin (flavonol glicoside)	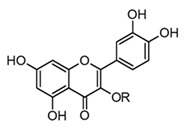 R = α-l-rhamnopyranosyl-(1→6)-β-d-glucopyranose	*In vivo* Mice–LPS	↓ CK and LDH ↑ Antioxidant enzymes (SOD) and (CAT) ↓ TNF-α and IL-6	*Tephrosia purpurea* *Citrus* spp. *Malus* spp. *Rubus* spp.	[57,66]
Luteolin (flavone)	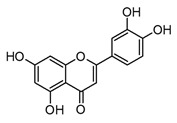	*In vitro* BMDM-LPS	↓ TNF-α and NF-κB ↓ iNOS expression ↓ IkB-α phosphorylation	*Capsicum frutescens* *Apium graveolens* *Garcinia* sp.	[67,69]
Diosmetin (flavone)	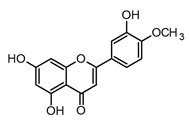	*In vitro* BMDM-LPS	↓ TNF-α and NF-κB ↓ iNOS expression ↓ IkB-α phosphorylation	*Citrus* spp. *Rosmarinus officinalis*	[58,69]
Pilloin (flavone)	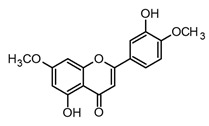	*In vitro* RAW 264.7-LPS *In vivo* septic mice–LPS	↓ TNF-α, IL-6, and COX-2 ↓ iNOS expression ↓ IkB-α phosphorylation JNK, ERK, and p38 inhibition	*Piper auritum* and *Murraya panaculata*	[70]
Pinocembrin (flavanone)	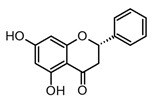	*In vitro* RAW 264.7-LPS BV2 microglia–LPS	↓ TNF-α, IL-6, and COX-2 ↓ iNOS and COX-2 expression ↓ TNF- α, IL-1β, NO, and PGE2 ↓ PI3K/Akt phosphorylation	*Piper* spp. *Peperomia* spp. Asteraceae family	[71,72]
Naringenin (flavanone)	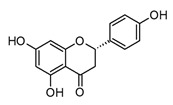	*In vitro* Murine macrophages–LPS *In vivo* Mouse–LPS	↓ NF-κB, PI3K/Akt, MAPK ↓ IL-4 and IL-13 ↓ Neutrophils and oxidative stress ↓ TNF and IL-6	*Citrus* spp. *Solanum lycopersicum*	[73]
Epigallocatechin-3-gallate EGCG (catechin)	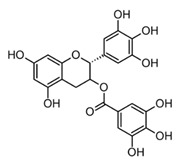	*In vitro* Human neutrophils–SOZ Human corneal cells–LPS	MPO inhibition ↓ HOCl and O_2_^−^. ↓ TNF-α, IL-1β, IL-6 and IL-8	*Camellia sinensis* L.	[74] (Li et al., 2020)

Abbreviations: BMDMs, bone marrow-derived macrophages; CAT, catalase; CK, creatine kinase; DSS, dextran sulfate sodium; IFN, interferon; IL, interleukin; IκB, inhibitor of NF-κB; JNK, c-Jun NH2-terminal kinase; LDH, lactate dehydrogenase; LPS, lipopolysaccharide; MAPK, mitogen-activated protein kinase; MPO, myeloperoxidase; NOS, nitric oxide synthase; NF-κB, nuclear factor–kappa B; PI3K, phosphatidylinositol 3-kinase; SOD, superoxide dismutase; SOZ, serum-opsonized zymosan; TNF, tumor necrosis factor. Cell lines: RAW 264.7 cells, murine macrophages derived from BALB/c mice; BV2 cells, murine microglial cell line derived from C57BL/6 mice. The value (expression or concentration) increased (↑) or decreased (↓).

**Table 3:** Selected polyphenols, structures, sources, and anti-inflammatory activity.

Compound (Class)	Structural Formula	Model/Assay	Effects and Mechanism	Sources in Tropical Plants	Ref.
Resveratrol (stilbene)	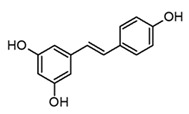	*In vivo* Balb-c mice Wistar rats	↓ TNF-α ↓ IL-10, IL-18, IL-6, and IL-1β ↓ iNOS and NO levels ↓ COX-2 expression	Gnetaceae Fabaceae Dipterocarpaceae Vitaceae Families	[83] (Dvorakova and Landa 2017)
Curcumin (curcuminoid)	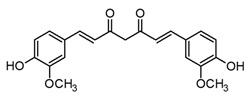	*In vitro* Human macrophages–LPS	↓ MMP1, MMP3, and MMP13 expression ↓ TNF-α inhibition of COX LOX production ↓ NO* and O_2_* levels ↓ iNOS expression	*Curcuma* genus	[82] (Rahaman et al., 2021)
Ferulic acid (phenylpropanoid)	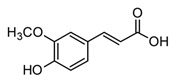	*In vitro* RAW 264.7-LPS	↓ TNF-α and IL-1β ↓ TNF-α and NF-κB	*Beta vulgaris* *Hordeum vulgare* *Zea mays everta*	[85] (Kwon et al., 2019)
Rosmarinic acid (phenylpropanoid ester)	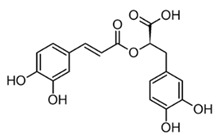	*In vitro* RAW 264.7-LPS *In vivo* Mice–LPS Lung injury	↓ TNF-α, IL-6, and IL-1β ↓ iNOS and NO levels ↓ TNF-α, IL-6, and IL-1β Inhibition of iNOS mRNA	Lamiacaeae family *Salvia* *Rosmarinus* *Mentha* *Occinmun* genus	[86] (Luo et al., 2020) [87] (Petersen 2013)

Abbreviations: IL, interleukin; LPS, lipopolysaccharide; MMP, matrix metalloproteinase gen; NO, nitric oxide; NOS, nitric oxide synthase; NF-κB, nuclear factor–kappa β; PGE_2_, prostaglandin E2; SOD, superoxide dismutase; TNF, tumor necrosis factor. Cell line: RAW 264.7, murine macrophages originally derived from BALB/c mice. The value (expression or concentration) decreased (↓).

**Table 4:** Selected examples of alkaloids, anti-inflammatory effects, and sources in tropical plants.

Compound (Sub-Class)	Structural Formula	Model/Assay	Effects and Mechanism	Sources in Tropical Plants	Ref.
Strictosidine (Indole)	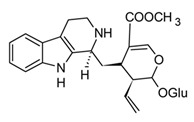	*In vitro* N9 cells–LPS	Inhibition of NO production ↓ COX-2 and iNOS expression	*Uncaria* genus *Catharanthus* spp.	[95] (Liang et al., 2020)
Mitraphylline (Oxindolic)	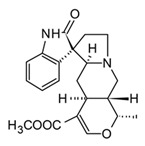	*In vivo* Balb/c female mice–LPS	↓ TNF-α, IL-1β, and IL-1α IL-17 and IL-4	*Uncaria tomentosa*	[96] (Rojas-Duran et al., 2012)
Mukolidine (Carbazole derivative)	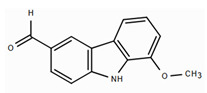	*In vitro* PBMCs-LPS *In vivo* Balb/c female mice–LPS	↓ TNF-α and IL-6 ↓ TNF-α and NF-κB	*Murraya* spp.	[97] (Nalli et al., 2016)
O-methylmurrayamine (Carbazole derivative)	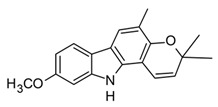	*In vitro* PBMCs-LPS *In vivo* Balb/c female mice–LPS	↓ TNF-α and IL-6 ↓ TNF-α and NF-κB	*Murraya* spp.	[97] (Nalli et al., 2016)
4-Methoxy-5-hydroxycanthin-6-one (β-carboline)	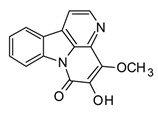	*In vitro* RAW 264.7-LPS	↓ TNF-α Inhibition of NO production ↓ iNOS expression	*Picrasma* spp.	[94] (Bai et al., 2021)
Harmine (β-carboline)	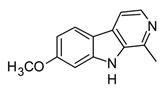	*In vitro* RAW 264.7-LPS *In vivo* Mice–LPS	↓ TNF-α, IL-1β and IL-6 NF-κB regulation	*Peganum* spp.	[94] (Bai et al., 2021)
Berberine (Protoberberine)	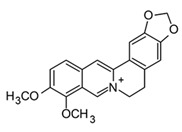	*In vitro* Macrophages–AcLDL	↓ TNF-α and IL-6 ↓ COX-2 expression ↓ NF-κB and MAPK signaling	*Berberis* spp. *Coptis* spp. *Cordalis* spp. *Zanthoxylum* spp.	[98] (Chen et al., 2008)
Glaucine (Aporphine)	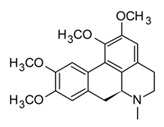	*In vitro* Macrophages–LPS Macrophages–zymosan	↓ TNF-α and IL-6 ↑ IL-10	*Glaucium flavum*	[99] (Remichkova et al., 2009)
Nitidine nornitidine norchelerythrine (benzophenanthridines)	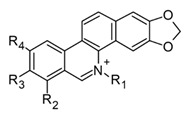 Nitidine: R_1_ = CH_3_, R_2_ = H, R_3_ = R_4_ = OCH_3_ Nornitidine: R_1_ = R_2_ = H, R_3_ = R_4_ = OCH_3_ Norchelerythrine: R_1_ = H, R_2_ = R_3_ = OCH_3_, R_4_ = H	*In vitro* 293 T cells RAW 264.7-LPS	↓ NF-κB ↓ TNF-α, IL-1β, and IL-6 ↓ iNOS expression	*Zanthoxylum* spp.	[94] (Bai et al., 2021)
Colchicine	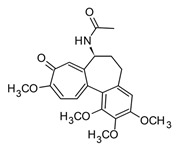	*In vitro* Macrophages–CPPD *In vivo*	↓ IL-1β Prevent TNF-α and IL-6	*Colchicum* spp.	[28]

Abbreviations: AcLDL, acetylated low-density lipoprotein; CPPD, calcium pyrophosphate dihydrate crystals; IL, interleukin; LPS, lipopolysaccharide; MAPK, mitogen-activated protein kinase; NO, nitric oxide; NOS, nitric oxide synthase; NF-κB, nuclear factor–kappa β; PGE_2_, prostaglandin E_2_; SOD, superoxide dismutase; TNF, tumor necrosis factor. Cell lines: N9 cells, murine microglial cell line derived from neonatal mouse brain and immortalized using v-myc; RAW 264.7, murine macrophages originally derived from BALB/c mice; 293T cells, human embryonic kidney cells from HEK 293 cells and transformed with the SV40 large T antigen. The value (expression or concentration) increased (↑) or decreased (↓).

## Data Availability

No new data were created or analyzed in this study. Data sharing is not applicable to this article.

## References

[1] Bloom D.E., Cadarette D. (2019). Infectious disease threats in the twenty-first century: Strengthening the global response. Front. Immunol..

[2] World Health Organization (2016). An R&D Blueprint for Action to Prevent Epidemics. Plan of Action.

[3] Mehand M.S., Al-Shorbaji F., Millett P., Murgue B. (2018). The WHO R&D Blueprint: 2018 Review of emerging infectious diseases requiring urgent research and development efforts. Antivir. Res..

[4] Liu Y.C., Kuo R.L., Shih S.R. (2020). COVID-19: The first documented coronavirus pandemic in history. Biomed. J..

[5] Andersen K.G., Rambaut A., Lipkin W.I., Holmes E.C., Garry R.F. (2020). The proximal origin of SARS-CoV-2. Nat. Med..

[6] Chaplin S. (2020). COVID-19: A brief history and treatments in development. Prescriber.

[7] World Health Organization (2020). Therapeutics and COVID-19. https://www.who.int/teams/health-care-readiness/covid-19/therapeutics.

[8] Alag S. (2020). Analysis of COVID-19 clinical trials: A data-driven, ontology-based, and natural language. PLoS ONE.

[9] Clinicaltrials.gov (2020). Clinical Trials: A Data-Driven, Ontology-Based, and Natural Language Processing Approach. CovidResearchTrials. https://clinicaltrials.gov/.

[10] GOV.UK (2020). UK Medicines Regulator Gives Approval for First UK COVID-19 Vaccine. https://www.gov.uk/government/news/uk-medicines-regulator-gives-approval-for-first-uk-covid-19-vaccine.

[11] FDA (2020). FDA Takes Key Action in Fight Against COVID-19 by Issuing Emergency Use Authorization for First COVID-19 Vaccine. https://www.fda.gov/news-events/press-announcements/fda-takes-key-action-fight-against-covid-19-issuing-emergency-use-authorization-first-covid-19.

[12] Khimani F., Wolf A.J., Yoon B., Blancke A., Gerhart C., Endsley D., Dougherty A., Ray A.K., Yango A.F., Flynn S.D. (2023). Therapeutic considerations for prevention and treatment of thrombotic events in COVID-19. Thromb. Update.

[13] Kimura-Ohba S., Asaka M.N., Utsumi D., Takabatake Y., Takahashi A., Yasutomi Y., Isaka Y., Kimura T. (2023). d-Alanine as a biomarker and a therapeutic option for severe influenza virus infection and COVID-19. Biochim. Biophys. Acta BBA-Mol. Basis Dis..

[14] Warrayat A., Ali A., Waked J., Tocci D., Speth R.C. (2024). Assessment of the therapeutic potential of salubrinal for ME/CFS and long-COVID. Trends Mol. Med..

[15] Song Y., Lu J., Qin P., Chen H., Chen L. (2024). Interferon-I modulation and natural products: Unraveling mechanisms and therapeutic potential in severe COVID-19. Cytokine Growth Factor Rev..

[16] Zhou Q., Zhang L., Dong Y., Wang Y., Zhang B., Zhou S., Huang Q., Wu T., Chen G. (2024). The role of SARS-CoV-2-mediated NF-κB activation in COVID-19 patients. Hypertens. Res..

[17] Hiti L., Markovič T., Lainscak M., Lainščak J.F., Pal E., Mlinarič-Raščan I. (2025). The Immunopathogenesis of a Cytokine Storm: The Key Mechanisms Underlying Severe COVID-19. Cytokine Growth Factor Rev..

[18] Yuki K., Fujiogi M., Koutsogiannaki S. (2020). COVID-19 pathophysiology: A review. Clin. Immunol..

[19] Vallamkondu J., John A., Wani W.Y., Ramadevi S.P., Jella K.K., Reddy P.H., Kandimalla R. (2020). SARS-CoV-2 pathophysiology and assessment of coronaviruses in CNS diseases with a focus on therapeutic targets. Biochim. Biophys. Acta Mol. Basis Dis..

[20] Hojyo S., Uchida M., Tanaka K., Hasebe R., Tanaka Y., Murakami M., Hirano T. (2020). How COVID-19 induces cytokine storm with high mortality. Inflamm. Regen..

[21] García L.F. (2020). Immune response, inflammation, and the clinical spectrum of COVID-19. Front. Immunol..

[22] Dias D.A., Urban S., Roessner U. (2012). A historical overview of natural products in drug discovery. Metabolites.

[23] Newman D.J., Cragg G.M., Snader K.M. (2000). The influence of natural products upon drug discovery. Nat. Prod. Rep..

[24] Azab A., Nassar A., Azab A.N. (2016). Anti-inflammatory activity of natural products. Molecules.

[25] Yuan G., Wahlqvist M.L., He G., Yang M., Li D. (2006). Natural products and anti-inflammatory activity. Asia Pac. J. Clin. Nutr..

[26] Parasher A. (2020). COVID-19: Current understanding of its pathophysiology, clinical presentation and treatment. Postgrad. Med. J..

[27] Zhang B., Zhou X., Zhu C., Song Y., Feng F., Qiu Y., Feng J., Jia Q., Song Q., Zhu B. (2020). Immune phenotyping based on neutrophil-to-lymphocyte ratio and IgG predicts disease severity and outcome for patients with COVID-19. Front. Mol. Biosci..

[28] Reyes A.Z., Hu K.A., Teperman J., Wampler Muskardin T.L., Tardif J.C., Shah B., Pillinger M.H. (2021). Anti-inflammatory therapy for COVID-19 infection: The case for colchicine. Ann. Rheum. Dis..

[29] Soy M., Keser G., Atagündüz P., Tabak F., Atagündüz I., Kayhan S. (2020). Cytokine storm in COVID-19: Pathogenesis and overview of anti-inflammatory agents used in treatment. Clin. Rheumatol..

[30] Hu B., Huang S., Yin L. (2021). The cytokine storm and COVID-19. J. Med. Virol..

[31] Del Valle D.M., Kim-Schulze S., Huang H.H., Beckmann N.D., Nirenberg S., Wang B., Lavin Y., Swartz T.H., Madduri D., Stock A. (2020). An inflammatory cytokine signature predicts COVID-19 severity and survival. Nat. Med..

[32] Kandasamy M. (2021). NF-ΚB signalling as a pharmacological target in COVID-19: Potential roles for IKKβ inhibitors. Naunyn Schmiedebergs Arch. Pharmacol..

[33] Harvey A., Edrada-Ebel R., Quinn R.J. (2015). The re-emergence of natural products for drug discovery in the genomics era. Nat. Rev. Drug Discov..

[34] Newman D.J., Cragg G.M. (2007). Natural products as sources of new drugs over the last 25 Years. J. Nat. Prod..

[35] Yongye A.B., Waddell J., Medina-Franco J.L. (2012). Molecular scaffold analysis of natural products databases in the public domain. Chem. Biol. Drug Des..

[36] Van Hattum H., Waldmann H. (2014). Biology-oriented synthesis: Harnessing the power of evolution. J. Am. Chem. Soc..

[37] Wang Q., Kuang H., Su Y., Sun Y., Feng J., Guo R., Chan K. (2013). Naturally derived anti-inflammatory compounds from Chinese medicinal plants. J. Ethnopharmacol..

[38] Maione F., Russo R., Khan H., Mascolo N. (2016). Medicinal plants with anti-inflammatory activities. Nat. Prod. Res..

[39] Napagoda M.T., Sundarapperuma T., Fonseka D., Amarasiri S., Gunaratna P. (2018). An ethnobotanical study of the medicinal plants used as anti-inflammatory remedies in Gampaha District, Western Province, Sri Lanka. Scientifica.

[40] Prasad S., Aggarwal B.B., Benzie I.F.F., Wachtel-Galor S. (2011). Turmeric, the Golden Spice: From Traditional Medicine to Modern Medicine. Herbal Medicine: Biomolecular and Clinical Aspects.

[41] Lantz R.C., Chen G.J., Solyom A.M., Jolad S.D., Timmermann B.N. (2005). The effect of turmeric extracts on inflammatory mediator production. Phytomedicine.

[42] Raven P.H., Gereau R.E., Phillipson P.B., Chatelain C., Jenkins C.N., Ulloa Ulloa C. (2020). The distribution of biodiversity richness in the tropics. Sci. Adv..

[43] Ribeiro V.P., Arruda C., Abd El-Salam M., Bastos J.K. (2018). Brazilian medicinal plants with corroborated anti- inflammatory activities: A review. Pharm. Biol..

[44] Alonso-Castro A.J., Juárez-Vázquez M.D.C., Campos-Xolalpa N. (2016). Medicinal plants from Mexico, Central America, and the Caribbean used as immunostimulants. Evid. Based Complement. Alternat. Med..

[45] Garrido G., González D., Lemus Y., García D., Lodeiro L., Quintero G., Delporte C., Núñez-Sellés A.J., Delgado R. (2004). *In vivo* and *in vitro* anti-inflammatory activity of *Mangifera indica* L. extract (VIMANG). Pharmacol. Res..

[46] Cosentino M., Bombelli R., Carcano E., Luini A., Marino F., Crema F., Dajas F., Lecchini S. (2008). Immunomodulatory properties of Achyrocline satureioides (Lam.) D.C. infusion: A study on human leukocytes. J. Ethnopharmacol..

[47] Ovalle-Marin A., Parra-Ruiz C., Rivas F., Orellana J.F., Garcia-Diaz D., Jimenez P. (2020). Characterization of *Persea americana* Mill. peels and leaves extracts and analysis of its potential *in vitro* anti-inflammatory properties. Boletín Latinoam. Caribe Plantas Med. Aromáticas.

[48] Gutierrez R., Hoyo-Vadillo C. (2017). Anti-inflammatory potential of *Petiveria alliacea* on activated RAW264.7 murine macrophages. Pharmacogn. Mag..

[49] Rivera D.E., Ocampo Y.C., Castro J.P., Barrios L., Diaz F., Franco L.A. (2019). A screening of plants used in Colombian traditional medicine revealed the anti-inflammatory potential of Physalis angulata calyces. Saudi J. Biol. Sci..

[50] De Morais Lima G.R., de Albuquerque Montenegro C., de Almeida C.L., de Athayde-Filho P.F., Barbosa-Filho J.M., Batista L.M. (2011). Database survey of anti-inflammatory plants in South America: A Review. Int. J. Mol. Sci..

[51] Abad M.J., Bessa A.L., Ballarin B., Aragón O., Gonzales E., Bermejo P. (2006). Anti-inflammatory activity of four Bolivian Baccharis species (Compositae). J. Ethnopharmacol..

[52] Gao T., Yao H., Song J., Liu C., Zhu Y., Ma X., Pang X., Xu H., Chen S. (2010). Identification of medicinal plants in the family Fabaceae using a potential DNA barcode ITS2. J. Ethnopharmacol..

[53] Veiga Junior V.F., Rosas E.C., Carvalho M.V., Henriques M.G., Pinto A.C. (2007). Chemical composition and anti-inflammatory activity of copaiba oils from Copaifera cearensis Huber Ex Ducke, Copaifera reticulata Ducke and Copaifera multijuga Hayne—A comparative study. J. Ethnopharmacol..

[54] Zhang H., Tsao R. (2016). Dietary polyphenols, oxidative stress and antioxidant and anti-inflammatory effects. Curr. Opin. Food Sci..

[55] Agati G., Azzarello E., Pollastri S., Tattini M. (2012). Flavonoids as antioxidants in plants: Location and functional significance. Plant Sci..

[56] Moccelini S.K., Da Silva V.C., Ndiaye E.A., De Sousa P.T., Vieira P.C. (2009). Phytochemical study from root barks of Zanthoxylum rigidum Humb. & Bonpl. ex Willd (Rutaceae). Quimica Nova.

[57] Panche A.N., Diwan A.D., Chandra S.R. (2016). Flavonoids: An overview. J. Nutr. Sci..

[58] Hostetler G.L., Ralston R.A., Schwartz S.J. (2017). Flavones: Food sources, bioavailability, metabolism, and bioactivity. Adv. Nutr..

[59] Vinayagam R., Xu B. (2015). Antidiabetic properties of dietary flavonoids: A cellular mechanism review. Nutr. Metab..

[60] Rodríguez-García C., Sánchez-Quesada C., Gaforio J. (2019). Dietary flavonoids as cancer chemopreventive agents: An updated review of human studies. Antioxidants.

[61] Teleanu R.I., Chircov C., Grumezescu A.M., Volceanov A., Teleanu D.M. (2019). Antioxidant therapies for neuroprotection-A review. J. Clin. Med..

[62] González J., García M.V., González-Gallego J., Sánchez S., Tuñón M.J., Watson R.R., Preedy V.R., Zibadi S. (2014). Anti-inflammatory and immunomodulatory properties of dietary flavonoids. Polyphenols in Human Health and Disease.

[63] Maleki S.J., Crespo J.F., Cabanillas B. (2019). Anti-inflammatory effects of flavonoids. Food Chem..

[64] Comalada M., Camuesco D., Sierra S., Ballester I., Xaus J., Gµlvez J., Zarzuelo A. (2005). *In vivo* quercitrin anti-inflammatory effect involves release of quercetin, which inhibits inflammation through down-regulation of the NF-KB pathway. Eur. J. Immunol..

[65] Karuppagounder V., Arumugam S., Thandavarayan R.A., Sreedhar R., Giridharan V.V., Watanabe K. (2016). Molecular targets of quercetin with anti-inflammatory properties in atopic dermatitis. Drug Discov. Today.

[66] Xianchu L., Lan Z., Ming L., Yanzhi M. (2018). Protective effects of rutin on lipopolysaccharide-induced heart injury in Mice. J. Toxicol. Sci..

[67] Miean K.H., Mohamed S. (2001). Flavonoid (myricetin, quercetin, kaempferol, luteolin, and apigenin) content of edible tropical plants. J. Agric. Food Chem..

[68] Hema K., Sukumar D. (2013). Isolation and phytochemical studies of quercetin and quercetin 3-O-rhamnoside. Int. J. Pharma. Biol. Sci..

[69] Comalada M., Ballester I., Bailon E., Sierra S., Xaus J., Gálvez J., Sánchez F., Zarzuelo A. (2006). Inhibition of pro-inflammatory markers in primary bone marrow-derived mouse macrophages by naturally occurring flavonoids: Analysis of the structure–activity relationship. Biochem. Pharmacol..

[70] Tsai Y.C., Wang S.L., Wu M.Y., Liao C.H., Lin C.H., Chen J.J., Fu S.L. (2018). Pilloin, a flavonoid isolated from *Aquilaria sinensis*, exhibits anti-inflammatory activity *in vitro* and *in vivo*. Molecules.

[71] Shen X., Liu Y., Luo X., Yang Z. (2019). Advances in biosynthesis, pharmacology, and pharmacokinetics of pinocembrin, a promising natural small-molecule drug. Molecules.

[72] Zhou L.T., Wang K.J., Li L., Li H., Geng M. (2015). Pinocembrin inhibits lipopolysaccharide-induced inflammatory mediators production in BV2 microglial cells through suppression of PI3K/Akt/NF-κB Pathway. Eur. J. Pharmacol..

[73] Alberca R.W., Teixeira F.M.E., Beserra D.R., De Oliveira E.A., Andrade M., Pietrobon A.J., Sato M.N. (2020). Perspective: The potential effects of naringenin in COVID-19. Front. Immunol..

[74] Li Z., Shi Z., Tang S., Yao H.P., Lin X., Wu F. (2020). Epigallocatechin-3-gallate ameliorates LPS-induced inflammation by inhibiting the phosphorylation of Akt and ERK signaling molecules in Rat H9c2 cells. Exp. Ther. Med..

[75] Jang S., Kelley K.W., Johnson R.W. (2008). Luteolin reduces IL-6 production in microglia by inhibiting JNK phosphorylation and activation of AP-1. Proc. Natl. Acad. Sci. USA..

[76] Liu Q., Ci X., Wen Z., Peng L. (2018). Diosmetin alleviates lipopolysaccharide-induced acute lung injury through activating the Nrf2 pathway and inhibiting the NLRP3 inflammasome. Biomol. Ther..

[77] Tutunchi H., Naeini F., Ostadrahimi A., Hosseinzadeh-Attar M.J. (2020). Naringenin, a flavanone with antiviral and anti-inflammatory effects: A promising treatment strategy against COVID-19. Phytother. Res..

[78] Martínez G., Mijares M.R., De Sanctis J.B. (2019). Effects of flavonoids and its derivatives on immune cell responses. Recent. Pat. Inflamm. Allergy Drug Discov..

[79] Zhang P., Mak J.C., Man R.Y., Leung S.W. (2019). Flavonoids reduces lipopolysaccharide-induced release of inflammmatory mediators in human bronchial epithelial cells: Structure-activity relationship. Eur. J. Pharmacol..

[80] Watson R.R., Preedy V.R., Zibadi S., Watson R., Preedy V., Zibadi S. (2018). Polyphenols: Prevention and Treatment of Human Disease.

[81] Gavrilas L.I., Cruceriu D., Ionescu C., Miere D., Balacescu O. (2019). Pro-apoptotic genes as new targets for single and combinatorial treatments with resveratrol and curcumin in colorectal cancer. Food Funct..

[82] Rahaman M.M., Rakib A., Mitra S., Tareq A.M., Emran T.B., Shahid-Ud-Daula A., Amin M.N., Simal-Gandara J. (2021). The genus curcuma and inflammation: Overview of the pharmacological perspectives. Plants.

[83] Dvorakova M., Landa P. (2017). Anti-inflammatory activity of natural stilbenoids: A review. Pharmacol. Res..

[84] Saqib U., Kelley T.T., Panguluri S.K., Liu D., Savai R., Baig M.S., Schürer S.C. (2018). Polypharmacology or promiscuity? Structural interactions of resveratrol with its bandwagon of targets. Front. Pharmacol..

[85] Kwon M.Y., Kim S.M., Park J., Lee J., Cho H., Lee H., Jeon C., Park J.H., Han I.O. (2019). A caffeic acid-ferulic acid hybrid compound attenuates lipopolysaccharide-mediated inflammation in BV2 and RAW264.7 cells. Biochem. Biophys. Res. Commun..

[86] Luo C., Zou L., Sun H., Peng J., Gao C., Bao L., Ji R., Jin Y., Sun S. (2020). A review of the anti-inflammatory effects of rosmarinic acid on inflammatory diseases. Front. Pharmacol..

[87] Petersen M. (2013). Rosmarinic acid: New aspects. Phytochem. Rev..

[88] Hewlings S.J., Kalman D.S. (2017). Curcumin: A review of its effects on human health. Foods.

[89] Zahedipour F., Hosseini S.A., Sathyapalan T., Majeed M., Jamialahmadi T., Al-rasadi K., Banach M., Sahebkar A. (2020). Potential effects of curcumin in the treatment of COVID-19 infection. Phytoter. Res..

[90] Panahi Y., Hosseini M.S., Khalili N., Naimi E., Majeed M., Sahebkar A. (2015). Antioxidant and anti-inflammatory effects of curcuminoid-piperine combination in subjects with metabolic syndrome: A randomized controlled trial and an updated meta-analysis. Clin. Nutr..

[91] Ortiz A., Sansinenea E. (2023). Phenylpropanoid Derivatives and Their Role in Plants’ Health and as antimicrobials. Curr. Microbiol..

[92] Fattorusso E., Taglialatela-Scafati O., Fattorusso E., Taglialatela-Scafati O. (2007). Modern Alkaloids: Structure, Isolation, Synthesis, and Biology.

[93] Aniszewski T. (2015). Alkaloids Chemistry, Biology, Ecology, and Applications.

[94] Bai R., Yao C., Zhong Z., Ge J., Bai Z., Ye X., Xie T., Xie Y. (2021). Discovery of natural anti-inflammatory alkaloids: Potential leads for the drug discovery for the treatment of inflammation. Eur. J. Med. Chem..

[95] Liang J.H., Wang C., Huo X.K., Tian X.G., Zhao W.Y., Wang X., Sun C.-P., Ma X.C. (2020). The genus Uncaria: A review on phytochemical metabolites and biological aspects. Fitoterapia.

[96] Rojas-Duran R., González-Aspajo G., Ruiz-Martel C., Bourdy G., Doroteo-Ortega V.H., Alban-Castillo J., Robert G., Auberger P., Deharo E. (2012). Anti-inflammatory activity of mitraphylline isolated from Uncaria tomentosa Bark. J. Ethnopharmacol..

[97] Nalli Y., Khajuria V., Gupta S., Arora P., Riyaz-Ul-Hassan S., Ahmed Z., Ali A. (2016). Four new carbazole alkaloids from Murraya koenigii that display anti-inflammatory and anti-microbial activities. Org. Biomol Chem..

[98] Chen F.L., Yang Z.H., Liu Y., Li L.X., Liang W.C., Wang X.C., Zhou W.B., Yang Y.H., Hu R.M. (2008). Berberine inhibits the expression of TNFa, MCP-1, and IL-6 in AcLDL-stimulated macrophages through PPARc Pathway. Endocrine.

[99] Remichkova M., Dimitrova P., Philipov S., Ivanovska N. (2009). Toll-like receptor-mediated anti-inflammatory action of glaucine and oxoglaucine. Fitoterapia.

[100] Cao R., Peng W., Wang Z., Xu A. (2007). β-Carboline alkaloids: Biochemical and pharmacological functions. Curr. Med. Chem..

[101] Liscombe D.K., MacLeod B.P., Loukanina N., Nandi O.I., Facchini P.J. (2005). Evidence for the monophyletic evolution of benzylisoquinoline alkaloid biosynthesis in angiosperms. Phytochemistry.

[102] Diamond A., Desgagné-Penix I. (2015). Metabolic engineering for the production of plant isoquinoline alkaloids. Plant Biotechnol. J..

[103] Sandjo L.P., Kuete V., Tchangna R.S., Efferth T., Ngadjui B.T. (2014). Cytotoxic benzophenanthridine and furoquinoline alkaloids from Zanthoxylum Buesgenii (Rutaceae). Chem. Cent. J..

[104] Bräse S. (2015). Privileged Scaffolds in Medicinal Chemistry.

[105] Plazas E., Casoti R., Avila Murillo M., Batista Da Costa F., Cuca L.E. (2019). Metabolomic profiling of Zanthoxylum species: Identification of anti-cholinesterase alkaloids candidates. Phytochemistry.

[106] Chu M., Chen X., Wang J., Guo L., Wang Q., Gao Z., Kang J., Zhang M., Feng J., Guo Q. (2018). Polypharmacology of berberine based on multi-target binding motifs. Front. Pharmacol..

[107] Yin J., Xing H., Ye J. (2008). Efficacy of berberine in patients with type 2 diabetes. Metabolism.

[108] Ehteshamfar S.M., Akhbari M., Afshari J.T., Seyedi M., Nikfar B., Shapouri-Moghaddam A., Ghanbarzadeh E., Momtazi-Borojeni A. (2020). Anti-inflammatory and immune-modulatory impacts of berberine on activation of autoreactive T cells in autoimmune inflammation. J. Cell Mol. Med..

[109] Di G., Xiang L.D., Yao S., Yu L.J., Da M.Z. (2009). A new benzophenanthridine alkaloid from Zanthoxylum Nitidum. Chin. J. Nat. Med..

[110] Slobodnick A., Shah B., Pillinger M.H., Krasnokutsky S. (2015). Colchicine: Old and new. Am. J. Med..

[111] Ludwiczuk A., Georgiev M.I. (2017). Chapter outline, and learning objectives. Terpenoids. Pharmacognosy.

[112] De las Heras B., Hortelano S. (2009). Molecular basis of the anti-inflammatory effects of terpenoids. Inflamm. Allergy Drug Targets.

[113] De Cássia da Silveira e Sá R., Andrade L.N., De Sousa D.P. (2013). A review on anti-inflammatory activity of monoterpenes. Molecules.

[114] Juergens U.R. (2014). Anti-inflammatory properties of the monoterpene 1,8-cineole: Current evidence for co-medication in inflammatory airway diseases. Drug Res..

[115] Jang H., Lee S., Lee S.J., Lim H., Jung K., Kim Y., Lee S., Rho M.C. (2017). Anti-Inflammatory activity of eudesmane-type sesquiterpenoids from Salvia plebeia. J. Nat. Med..

[116] Chen X., Zong C., Gao Y., Cai R., Fang L., Lu J., Liu F., Qi Y. (2014). Curcumol exhibits anti-inflammatory properties by interfering with the JNK-mediated AP-1 pathway in lipopolysaccharide-activated RAW264.7 cells. Eur. J. Pharmacol..

[117] Mathema V.B., Koh Y.S., Thakuri B.C., Sillanpää M. (2012). Parthenolide, a sesquiterpene lactone, expresses multiple anti-cancer and anti-Inflammatory activities. Inflammation.

[118] Li L., Wei S., Zhu T., Xue G., Xu D., Wang W., Wang X., Luo J., Kong L. (2019). Anti-inflammatory norabietane diterpenoids from the leaves of *Salvia officinalis* L.. J. Funct. Foods..

[119] Jeong G.S., Bae J.S. (2014). Anti-inflammatory effects of triterpenoids; naturally occurring and synthetic agents. Mini Rev. Org. Chem..

[120] Chadwick M., Trewin H., Gawthrop F., Wagstaff C. (2013). Sesquiterpenoids lactones: Benefits to plants and people. Int. J. Mol. Sci..

[121] Kwok B.H., Koh B., Ndubuisi M.I., Elofsson M., Crews C.M. (2001). The anti-infammatory natural product parthenolide from the medicinal herb feverfew directly binds to and inhibits IkB kinase. Chem. Biol..

[122] Bahrami M., Kamalinejad M., Latifi S.A., Seif F., Dadmehr M. (2020). Cytokine storm in COVID-19 and parthenolide: Preclinical evidence. Phytother. Res..

[123] Han N., Bakovic M. (2015). Biologically active triterpenoids and their cardioprotective and anti-inflammatory effects. J. Bioanal. Biomed..

